# Single-shot photoacoustic imaging with single-element transducer through a spatiotemporal encoder

**DOI:** 10.1117/1.JBO.28.4.046004

**Published:** 2023-04-13

**Authors:** Yanyu Zhao, Lihong V. Wang

**Affiliations:** California Institute of Technology, Caltech Optical Imaging Laboratory, Andrew and Peggy Cherng Department of Medical Engineering, Pasadena, California, United States

**Keywords:** single-shot imaging, compressive imaging, photoacoustic imaging, spatiotemporal encoding

## Abstract

**Significance:**

Current photoacoustic (PA) imaging modalities typically require either serial detection with a single-element transducer or parallel detections with an ultrasonic array, indicating a dilemma between system cost and imaging throughput. PA topography through ergodic relay (PATER) was recently developed to address this bottleneck. However, PATER requires object-specific calibration due to varied boundary condition and must be recalibrated through pointwise scanning for each object before measurements, which is time-consuming and severely limits practical application.

**Aim:**

We aim to develop a new single-shot PA imaging technique that only requires a one-time calibration for imaging different objects using a single-element transducer.

**Approach:**

We develop an imaging method, PA imaging through a spatiotemporal encoder (PAISE), to address the above issue. The spatial information is effectively coded into unique temporal features by the spatiotemporal encoder, which allows for compressive image reconstruction. An ultrasonic waveguide is proposed as a critical element to guide the PA waves from the object into the prism, which effectively accounts for the varied boundary condition of different objects. We further add irregular-shaped edges on the prism to introduce randomized internal reflections and further facilitate the scrambling of acoustic waves.

**Results:**

The proposed technique is validated through comprehensive numerical simulations and experiments, and it is demonstrated that PAISE can successfully overcome the changed boundary condition and can image different samples given a single calibration.

**Conclusions:**

The proposed PAISE technique is capable of single-shot widefield PA imaging with a single-element transducer and does not require sample-specific calibration, which successfully overcomes the major limitation of previous PATER technology.

## 1 Introduction

Optical imaging reveals important molecular and structural information in biological tissues. Microscopy technologies, such as confocal microscopy, multiphoton microscopy, and optical coherence tomography, can form high-resolution images through millimeter depth by detecting backscattered or fluorescent photons.^1^^–^^4^ Diffuse optical imaging technologies, such as diffuse optical tomography and spatial frequency-domain imaging, can probe deeper in biological tissues at the cost of lower resolution due to the nature of photon diffusion.^5^^–^^7^ As a complementary tool, photoacoustic (PA) imaging has a unique advantage of penetrating centimeters in biological tissues while maintaining high spatial resolution. With sensitivity toward optical absorption, PA imaging can probe both molecular and structural information, which holds promise for a wide range of applications.^8^^–^^11^

To date, most PA imaging systems require either point-by-point scanning using a single-element ultrasonic transducer or parallel detections using an ultrasonic transducer array. While the former is typically time-consuming and has a limited imaging throughput, the latter is complex and expensive.^11^^–^^15^

Here, we propose a low-cost high-throughput PA imaging technique enabled by a spatiotemporal encoder (SE) to image different objects with only a single-element ultrasonic transducer and a single laser shot and without the need of object-specific calibration. We refer to this technique as PA imaging through a spatiotemporal encoder (PAISE). Previously, Li et al. demonstrated the feasibility of PA imaging with a single-element transducer [i.e., PA topography through ergodic relay (PATER)]. However, PATER requires object-specific calibration for each imaging object. In other words, the system must be recalibrated through pointwise scanning for each object before the measurement, which is time-consuming and severely limits practical application.^16^ In contrast, PAISE is featured with object-independent calibration, and once calibrated, can be applied for single-shot imaging of different objects, which overcomes the major limitation of PATER technology.

## 2 Photoacoustic Imaging Through a Spatiotemporal Encoder

**Fig. 1:**
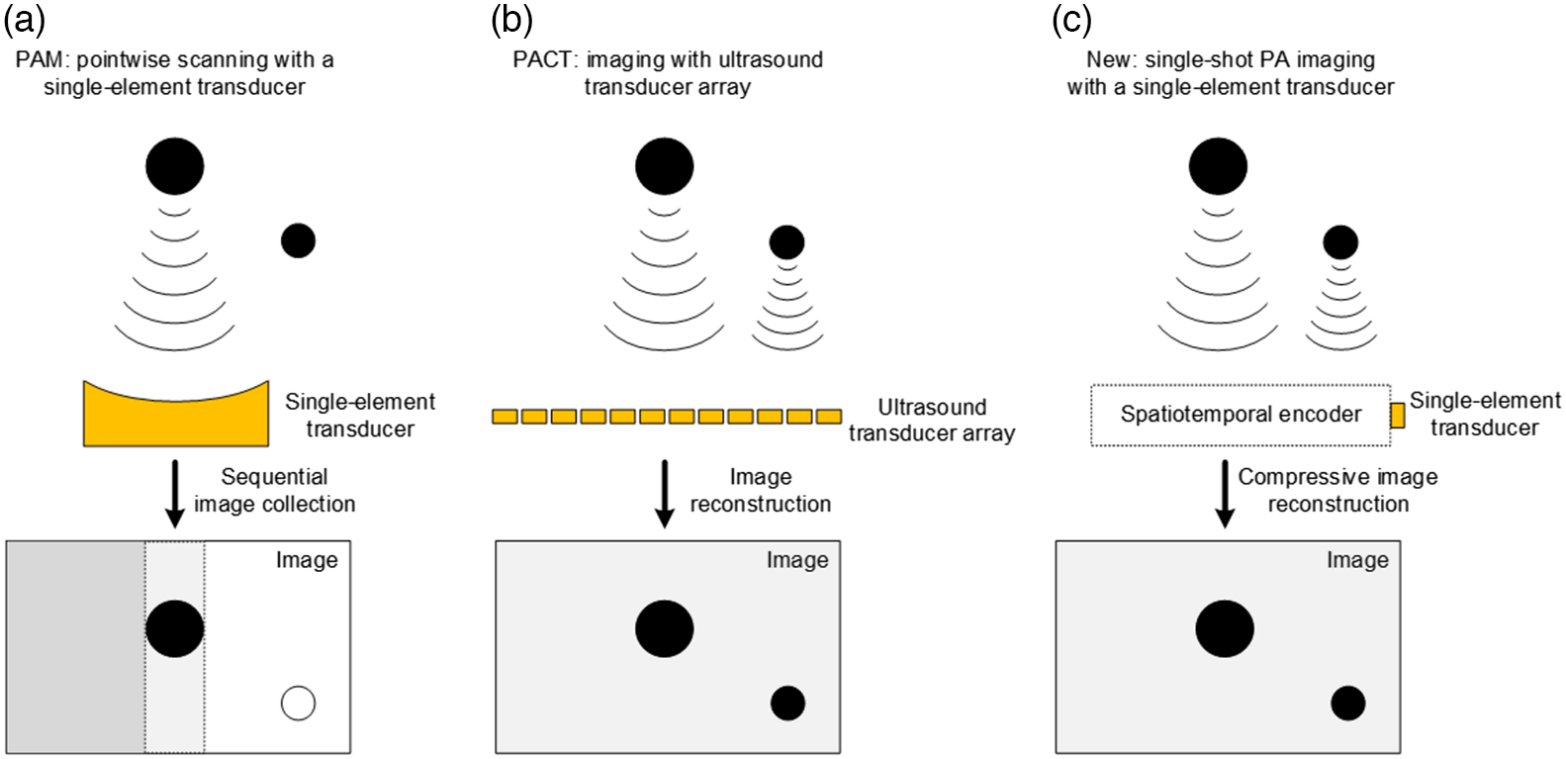
Three formations of PA imaging. (a) Conventional PA microscopy utilizes a single-element transducer but requires pointwise scanning to form an image. (b) PACT applies widefield illumination and detects PA waves in parallel, but it requires ultrasound transducer array which is complex and expensive. (c) With an SE, the proposed PAISE is capable of single-shot widefield imaging using a single-element transducer without the need of object-specific calibration.

PAISE differs from conventional PA imaging in terms of measurement mechanism and image reconstruction. On the one hand, PA microscopy (such as OR-PAM) focuses a short light pulse onto a small spot (e.g., a diffraction-limited point) of the object to be imaged. The illuminated spot absorbs light energy and converts it into heat and consequently emits PA wave due to thermoelastic expansion.^17^^,^^18^ Subsequently, a single-element focused transducer detects the PA wave. The object image is eventually acquired through pointwise scanning of the entire field-of-view (FOV), as shown in Fig. 1(a). On the other hand, as shown in Fig. 1(b), photoacoustic computed tomography (PACT) applies widefield illumination upon the imaging object and uses a transducer array to detect emitted PA waves from the entire FOV in parallel. The detected signals are then used to reconstruct the object image. In contrast, PAISE (as well as PATER) uses a single-shot widefield illumination and only a single-element transducer to capture the entire image without scanning. As shown in Fig. 1(c), the PA waves from the FOV are encoded by the SE. The encoded signal is recorded by the single-element transducer, and the image is recovered through compressive image reconstruction.

**Fig. 2:**
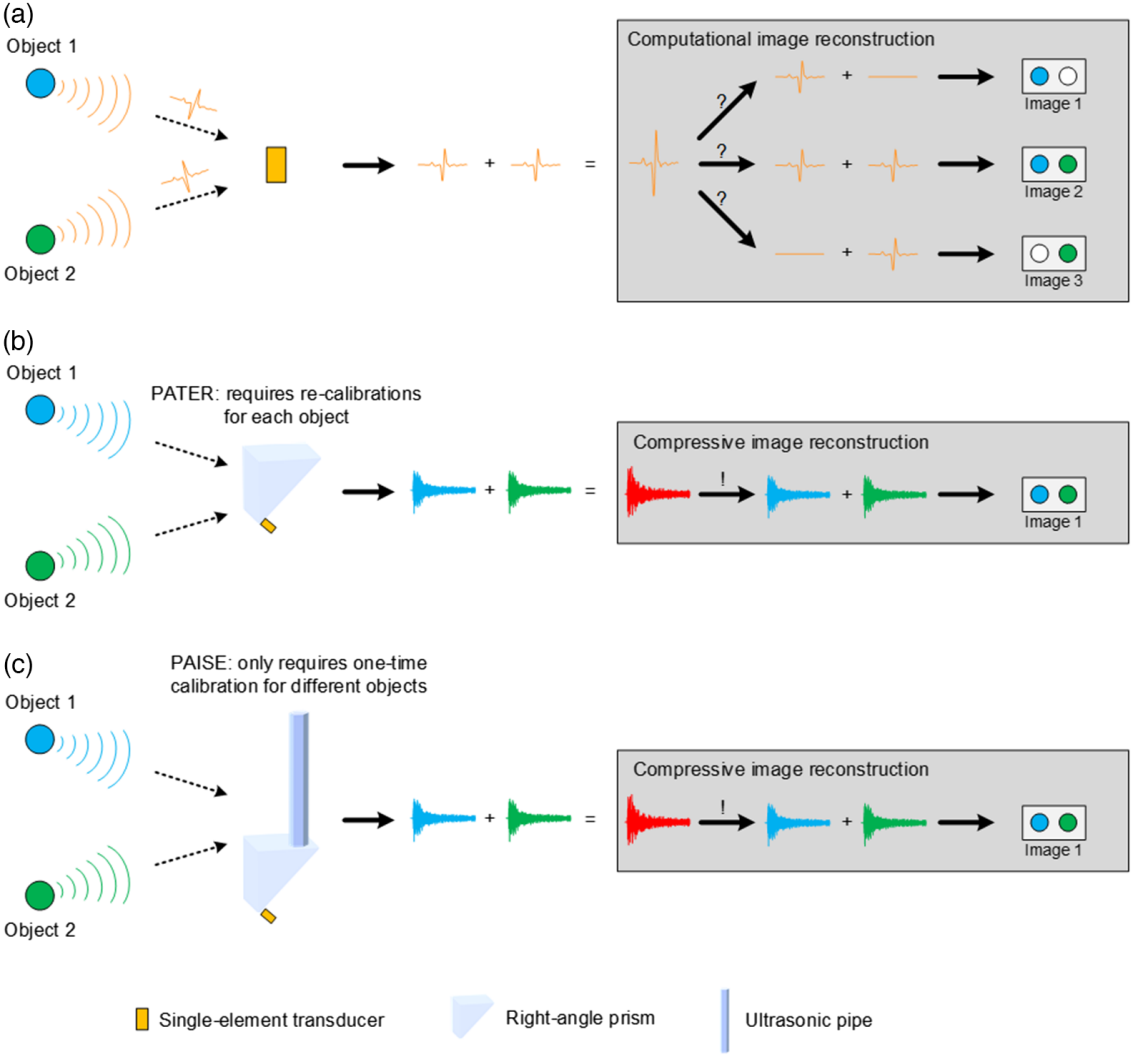
An SE allows for single-shot widefield imaging with single-element transducer. (a) A single-element transducer cannot distinguish objects at symmetric locations. (b) With a right-angle prism, previous PATER technique is capable of signal separation between two objects but requires object-specific calibrations. (c) The proposed PAISE is capable of unique signal separation between two objects through compressive image reconstruction and only requires a one-time calibration for different objects.

To achieve compressive image reconstruction, the information of different spatial locations needs to be projected onto a set of incoherent basis functions via spatiotemporal encoding. As shown in Fig. 2(a), given a single-element transducer and two objects at its symmetric locations, the transducer can detect PA waves from both objects, but is unable to distinguish the source of the signal. In PATER, a right-angle prism was proposed to break the symmetry. As shown in Fig. 2(b), the PA waves from the objects contain unique temporal features and can be distinguished from the combined signal through compressive image reconstruction. However, it requires pointwise calibration of the entire FOV for each new object, which is time-consuming and severely limits practical application. To address the bottleneck of object-specific calibration, we propose a spatiotemporal encoding strategy (i.e., PAISE), which features object-independent calibration. As shown in Fig. 2(c), PAISE uses an ultrasonic pipe combined with a right-angle prism to achieve unique spatiotemporal encoding of PA waves originating from different locations in the FOV. In addition, the ultrasonic pipe is placed at the corner of the right-angle prism, so the ultrasonic signals from the object can be sufficiently scrambled (i.e., encoded) in the time domain to facilitate image reconstruction.

## 3 Propagation of PA Waves in the Spatiotemporal Encoder

**Fig. 3:**
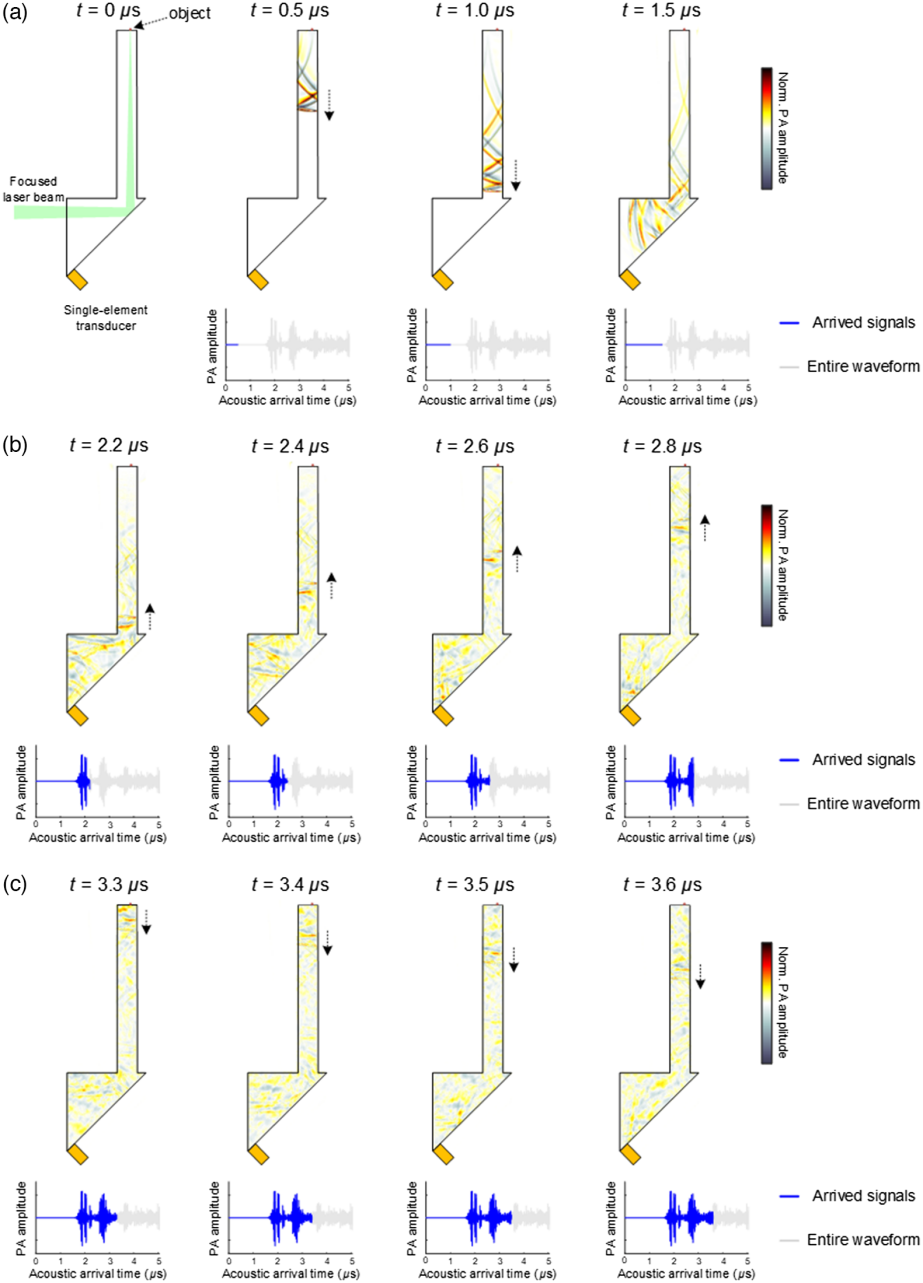
Propagation of PA waves in the SE. (a) The generated PA wave travels along the ultrasonic pipe toward the right-angle prism. (b) The PA wave reverberates inside the prism, and part of the wave travels back into the ultrasonic pipe toward the object. (c) The reflected ultrasound wave, which is dependent on the boundary condition, travels back into the prism. The blue curves represent arrived signal at different time points, and the gray curves represent the entire waveform.

The key advantage of PAISE over PATER is that it does not require object-specific calibration. In other words, once calibrated, PAISE can be applied to different objects to achieve single-shot PA imaging with a single-element transducer. To illustrate the mechanism of such advantage, the propagation of PA waves in the proposed SE is simulated based on the k-wave toolbox,^19^ and representative ultrasound wave fields are shown in Fig. 3. In Fig. 3, the simulation was conducted in two-dimensional (2D) to give a side view of the propagation of PA waves. The ultrasonic pipe and the right-angle prism were both modeled as fused silica (with 5900  m/s speed of sound), and the surrounding medium was modeled as air (with 343  m/s speed of sound). To aid the visualization of the propagation of PA waves in the pipe and the prism, the lengths were set as 6 cm for the pipe and 3 cm for the prism. In addition, the voxel size was set as $0.1\;{\mathrm{mm}}^{3}$ for the simulation. As shown in Fig. 3(a), the object is placed on top of the ultrasonic pipe, and the single-element transducer is placed at the corner of the right-angle prism. At t=0  μs, the short light pulse illuminates the object and generates PA wave. The ultrasound wave fields from timepoint 0.5 to 1.5  μs clearly illustrate the propagation of the PA wave traveling from the object, through the ultrasound pipe, and into the right-angle prism. The recorded PA signal is also plotted, and the blue curves represent the arrived signal at different time points, while the entire waveform is shown in gray. The flat blue lines in Fig. 3(a) indicate that until t=1.5  μs, the PA wave has not yet arrived at the transducer. After arrival into the prism, the PA wave bounces within the prism and at the same time the transducer records a unique time series which encodes the spatial information of the source of the PA wave. It is noted that while the PA wave bounces inside the prism, part of the wave would find its way back into the ultrasonic pipe and travel toward the object, as shown in Fig. 3(b). When the ultrasound wave reaches the top of the pipe, it would interact with the boundary (including the original object) and then travel back toward the prism, as shown in Fig. 3(c). It is important to note that the ultrasound wave coming back from the top boundary is dependent on the boundary condition, and after entering the prism, would affect the temporal feature of the recorded signal. On the other hand, the signals record before the arrival of the reflected PA wave is independent from the boundary condition. Such mechanism is the key feature that enables object-independent calibration in PAISE. In contrast, note that in PATER, the recorded signal would be continuously affected by the boundary condition, which dictates recalibrations for each imaging object.

## 4 Experimental Validations

### 4.1 Comparison of Image Reconstruction Between PATER and PAISE

To further illustrate the capability of object-independent calibration, we compared the proposed PAISE against PATER for image reconstruction with numerical simulations.

Following the procedures described in Li et al.,^16^ the compressive image reconstruction was implemented with a two-step iterative shrinkage/thresholding (TwIST) algorithm.^20^ Specifically, the signal recorded on the single-element transducer, denoted as s, can be represented as a linear combination of the calibration basis, denoted as $k_{i}$, as shown in Eq. (1):

$$s=\overset{N}{\underset{i=1}{\sum }}k_{i}P_{i},$$ (1)

where i, N, and $P_{i}$ denote calibrated pixel index, the total number of calibrated pixel locations, and the pixel intensity of the widefield image, respectively. Equation (1) can be cast to a matrix form for simplicity: s=KP, where $K=[k_{1},k_{2},\ldots ,k_{N}]$ and $P=[P_{1},P_{2},\ldots ,P_{N}]$. The image P can be recovered by minimizing the objective function in Eq. (2), where $\Phi_{\mathrm{TV}}(P)$ is the total variation regularization term, and λ is the regularization parameter:

P^=arg minP ‖s−KP‖2+2λΦTV(P). (2)

**Fig. 4:**
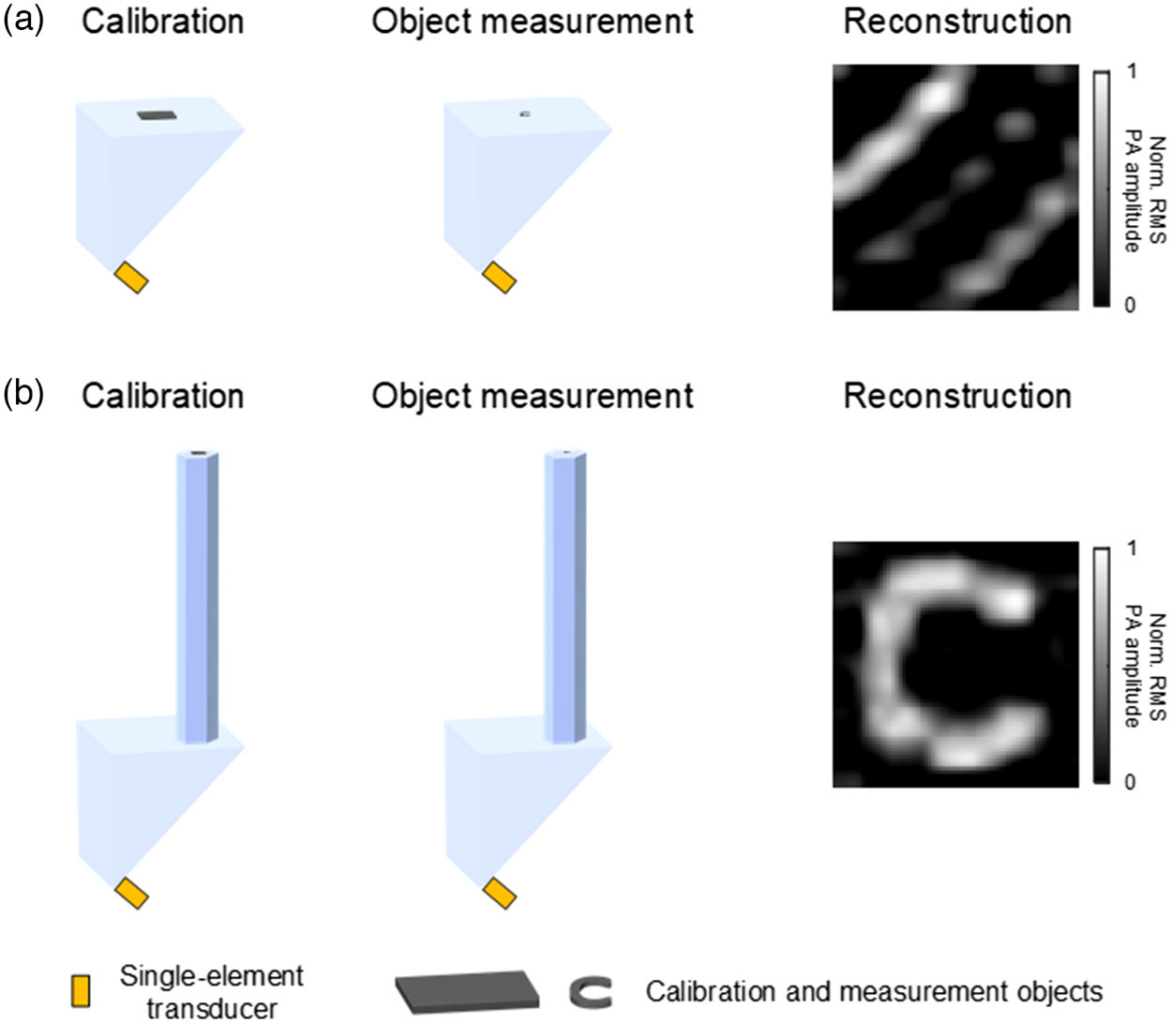
Comparison of PATER and PAISE for image reconstruction. (a) After pointwise calibration, PATER was not able to image new object due to changed boundary condition between the calibration and measurement objects. (b) After calibration, the proposed PAISE successfully recovered the image of the new object regardless of the changed boundary condition.

In Fig. 4, to compare the image reconstruction between PATER and PAISE, the simulation was conducted in 3D. In the simulation, the prism was modeled as an off-the-shelf right-angle prism. Different from 2D simulation, 3D simulation requires significantly more voxels and consequently challenges the RAM size of the PC as well as CPU runtime. As proof-of-concept, in the 3D simulation (with voxel size of $0.1\;{\mathrm{mm}}^{3}$), the lengths of the pipe and the prism were set as 30 and 5 mm, respectively. The transducer was set as 0.2 mm in size and placed in direct contact with the lower corner of the prism. In addition, we note that the size and position of the transducer can be moderately adjusted without affecting the calibration, but one can envision that if the transducer is as large as the prism, the calibration would not work very well, since the acoustic waves would not be able to sufficiently scramble inside the prism before being collected by the transducer.

As shown in Fig. 4, first, the calibration object was placed on top of the right-angle prism and pointwise calibration was conducted. Then, a C-shaped object was placed on the prism for widefield measurement. As shown in Fig. 4(a), the PATER was not able to reconstruct the C-shaped object due to the changed boundary condition between the calibration and the measurement. In contrast, with the proposed PAISE, the C-shaped object was successfully recovered in image reconstruction, as shown in Fig. 4(b).

### 4.2 Single-Shot Imaging of Different Objects with a Single Calibration

In addition to the numerical simulation, the proposed PAISE was further validated in experiment to demonstrate single-shot imaging using a single-element transducer with a single calibration.

A combination of customized right-angle prism (PS611, Thorlabs, Inc.; 2.5 cm right-angle edge length) and an ultrasonic pipe (#65-839, Edmund Optics; 1 cm hexagonal aperture, 17.5 cm length) was chosen to serve as the SE. Both the prism and the ultrasonic pipe were made of UV-fused silica, which had negligible attenuation over the acoustic detection pathlength. A custom-made unfocused pin transducer (30 MHz central frequency and 1 mm element size) was placed at the corner of the prism to collect ultrasound signal. To facilitate ultrasound transmission, ultrasound gel was applied between the object and the pipe, while a thin layer of polyester resin was added between the prism and the pin transducer. We note that although polyester resin would generally impede ultrasound transmission, counterintuitively, we found a thin layer of it to be very helpful in our experiment: since it does not dry over time, one would not need to add the coupling gel and position the transducer repeatedly. In addition, a nanosecond laser with 532 nm wavelength and 5 ns pulse width (INNOSAB IS8II-E, EdgeWave GmbH; 2 kHz repetition rate) was used as light source in the PAISE system. The laser beam was partially reflected by a beam splitter to a photodiode (DET36A, Thorlabs, Inc.) for the correction of fluctuations in pulse energy. In calibration, the laser beam was focused through a lens (LA1433-A, Thorlabs, Inc.; 2.54 cm diameter and 150 mm focal length) onto the calibration object for pointwise scanning. A motorized XY scanner (PLS-85, Physik Instrumente, Germany) was used for calibration in our experiment. Alternatively, a galvo-resonant scanner can also be utilized for improved scanning speed. In widefield measurement, the laser beam passed through an engineered diffuser (EDC-5-A-1r, RPC Photonics, Inc.; 5.5 deg divergence angle) to provide widefield illumination over the entire FOV. For data acquisition, a two-channel digitizer (ATS9350, AlazarTech, Inc.; 12 bits, 250  MS/s sampling rate, and 65,536 data points/acquisition sampling length) was used to record the PA signal and the photodiode output. The calibration and measurement objects were made of black rubber.

**Fig. 5:**
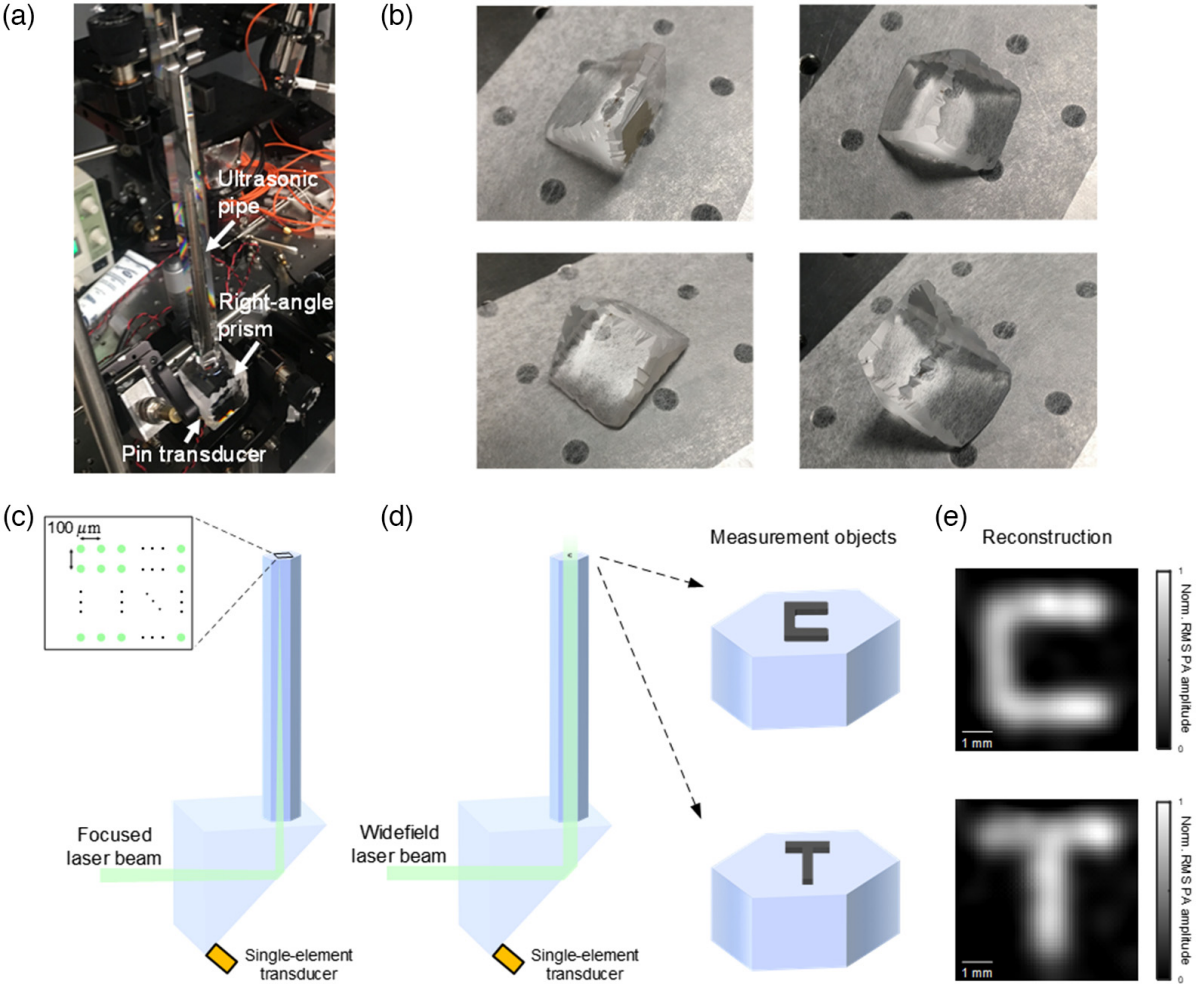
Validation of PAISE with different measurement objects. (a) Picture of key experimental setup including the ultrasonic waveguide, right-angle prism, and pin transducer. (b) Pictures of the customized right-angle prism from different viewing-angles showing irregular-shaped edges. (c) The system was calibrated by pointwise scanning using a focused laser beam. The calibrated area was 6×6  mm and the scanning increments were 100  μm. (d) After calibration, a C-shaped object and a T-shaped object were placed on top of the ultrasonic pipe and measured with widefield laser beam. (e) Both objects were successfully recovered in the image reconstruction.

Figure 5(a) shows a picture of the key experimental setup, including the ultrasonic waveguide, right-angle prism, and pin transducer. While PATER used an off-the-shelf right-angle prism, in our experiment we added irregular-shaped edges on the right-angle prism (using a grinder) to introduce randomized internal reflections and further facilitate the scrambling of acoustic waves. We note that the customized right-angle prism was not used in simulation due to the challenges of accurate modeling of its irregular-shaped edges. In addition, to make such a customized prism, one simply needs to randomly grind (for example, use a bench grinder and carefully hold in hand when grinding to introduce randomness) groove/ridge patterns onto the original edges of the off-the-shelf right-angle prism, where the groove/ridge patterns should be considerably larger than the acoustic wavelength to allow reflection. We note that while both the off-the-shelf right-angle prism and the customized right-angle prism can achieve object-independent calibration (when combined with the ultrasonic waveguide), the customized prism has the benefits of improved resolution and image reconstruction. Pictures of the customized right-angle prism from different viewing-angles are shown in Fig. 5(b) to illustrate the irregular-shaped edges. In experiment, as shown in Fig. 5(c), the calibration object was placed on top of the ultrasonic pipe, and a focused laser beam was used to scan a 6×6  mm FOV on the object with 100-μm increments. For widefield measurement, as shown in Fig. 5(d), the calibration object was removed, and a C-shaped object was placed inside the calibrated FOV on top of the ultrasonic pipe. A broad laser beam was sent to illuminate the object and the PA signal was collected. Then, another widefield measurement was conducted for a T-shape object. As shown in Fig. 5(e), both objects were successfully recovered in the image reconstruction. The results demonstrated that once calibrated, the proposed PAISE was able to image different objects regardless of the changed boundary condition. In terms of time cost, the image reconstruction with TwIST algorithm took 3.6±0.1  s (repeated 10 times) using a desktop computer with Intel Xeon Silver 4210R CPU (2.40 GHz) and 128 GB RAM. In the future, the reconstruction process can potentially be significantly accelerated by developing a deep learning model that does not involve iterative optimization and can directly output the reconstructed image in a straightforward manner.^21^^–^^24^

In addition, we note that the same experiments were also conducted for PATER. As expected, PATER was not able to reconstruct different objects after a single calibration due to changed boundary conditions (data not shown).

## 5 Discussion

We have developed and validated PAISE as a new method for single-shot PA imaging with single-element transducer. This was enabled through the use of an SE, which was able to effectively transform spatial information into unique temporal features. The ultrasonic waveguide in combination with a right-angle prism allows for separation of varied boundary conditions from encoded PA signals. In comparison to the conventional PA technologies, we demonstrated that the proposed PAISE was able to translate encoded temporal signature into original spatial locations using a single transducer. We further compared the proposed PAISE against PATER through comprehensive simulations to demonstrate the advantage of object-independent calibration. Finally, we validated the PAISE in experiments and demonstrated single-shot imaging of different objects without the need of object-specific calibration.

PAISE enjoys a number of advantages over current PA modalities. Compared with popular PA microscopy techniques that also uses a single-element transducer, PAISE is able to perform single-shot imaging, while PA microscopy techniques require pointwise scanning over the entire FOV. PACT is able to conduct single-shot imaging and does not require pointwise scanning. However, PACT requires an ultrasonic transducer array and is known for being complex and expensive, whereas PAISE only requires a single-element transducer.^11^^,^^14^ In addition, while PATER can also conduct single-shot imaging using a single-element transducer, it requires recalibration for each measurement object due to varied boundary conditions. In contrast, PAISE only requires one-time calibration and can be applied to different measurement objects without recalibration. Furthermore, while PATER is limited for 2D imaging, it is important to note that PAISE can potentially achieve single-shot 3D imaging. This is because the depth information is intrinsically encoded in the time of arrival of the acoustic signal, and the 3D image of different objects can subsequently be recovered given calibration of the 3D volume.

Regarding the pipe selection, we would like to note that to address the limitation of PATER (i.e., repeated calibration for different objects), we took significant amount of time to explore various potential geometries of a new SE, such as rectangular ones, curved ones, triangular ones, etc. In the end, we found the presented version (i.e., a pipe on top of a right-angle prism) to be a very simple yet effective solution. In terms of parameter selection, the diameter of the pipe should be significantly smaller than the length of the right-angle prism, so the ultrasonic signals from the object can be effectively scrambled and encoded in the time domain. In addition, the length of the pipe should be much longer compared to the length of the right-angle prism. This is critical, since after the PA signal from the object arrives the right-angle prism, while it gets partially reflected back into the pipe for a round trip, the remaining PA signal, without being affected by the changed boundary condition, requires a sufficient time window to find its way to the transducer through multiple internal reflections. Essentially, the duration of that time window is determined by the length of the pipe (i.e., time for the round trip).

In terms of the shape of the utilized transducer, in principle, one can use both flat and curved transducers in the experiment. In the presented setup, the flat transducer can be placed in contact with the prism (through a thin layer of polyester resin) and efficiently record PA signals. In contrast, if using a curved transducer, such as a focused transducer, one would have to add more coupling materials between the transducer and the prism surface, which may affect the SNR of the recorded signals.

In terms of FOV, PATER demonstrated $8\times 6\;{\mathrm{mm}}^{2}$ FOV while the proposed PAISE demonstrated $6\times 6\;{\mathrm{mm}}^{2}$ FOV. The FOV of the proposed method can be extended using an ultrasonic pipe with a larger aperture. In addition, it is worth noting that the FOV of both methods can be extended by calibrating a larger area.

In terms of resolution, similar to PATER, the resolution limit of the proposed system is also fundamentally determined by the acoustic wavelength in the material of the encoder (e.g., fused silica) at the ultrasonic transducer’s central frequency. In addition to the in-depth analysis of imaging resolution included in the previous PATER work, we would like to add one more note regarding the transducer and the prism for optimizing resolution in experiment. To achieve optimized resolution, after entering the prism, the acoustic waves should be sufficiently scrambled, so the spatial information of the object can be effectively transformed (i.e., encoded) into unique temporal features of the collected time series. Specifically, on the one hand, the size of the transducer should be relatively small so the acoustic waves can be sufficiently scrambled in the SE before being collected by the transducer. On the other hand, if the size of the transducer is too small, only a tiny fraction of the acoustic waves can be collected, which would lead to deteriorated SNR and consequently decreased resolution. In our study, we found the 1 mm transducer to be feasible in experiment. In addition, the shape of the prism is also crucial for the resolution. In experiment, we found that when using an off-the-shelf right-angle prism, while achieving object-independent calibration, the system resolution was approximately the size of the transducer. To push the resolution toward the acoustic wavelength, in this study, we added irregular-shaped edges on the prism to introduce randomized internal reflections and further facilitate the scrambling of acoustic waves. The customized prism enabled resolution close to 0.2 mm (data not shown), which is significantly smaller than the size of the transducer.

This work has several important implications for scientific research and clinical applications. For example, brain cortex imaging is of fundamental significance for neuroscience.^5^^,^^25^^–^^28^ Current methods such as two-photon microscopy and fluorescence imaging are typically limited by a small FOV and requires exogenous contrast agents. In contrast, the proposed PAISE can potentially provide both large FOV and label-free functional imaging. Furthermore, in terms of temporal resolution, given sufficient repetition rate of the light source, PAISE can potentially achieve kilohertz monitoring, which is similar to that of EEG methods,^16^ while the latter is known to have limited spatial resolution. Therefore, in the future, PAISE may be a powerful tool for brain science research. With regard to clinical applications, PAISE may also enable single-shot stain-free histopathology combined with UV wavelengths, which would be significantly faster than current UV-PAM modalities that requires pointwise scanning.^15^^,^^29^^,^^30^

In summary, we have developed PAISE, a new imaging modality that can conduct single-shot imaging using a single-element transducer without the need of object-specific calibration, which successfully overcomes the major limitation of previous PATER technology. In the future, PAISE can be potentially useful for a wide range of biomedical applications.
